# Achromatic and chromatic contrast discrimination in patients with type 2 diabetes

**DOI:** 10.1038/s41598-023-34407-1

**Published:** 2023-05-08

**Authors:** Li-Ting Tsai, Chien-Chung Chen, Chiun-Ho Hou, Kuo-Meng Liao

**Affiliations:** 1 School of Occupational Therapy, College of Medicine, National Taiwan University, Taipei, Taiwan; 2 Department of Ophthalmology, National Taiwan University Hospital, Taiwan College of Medicine, National Taiwan University, Taipei, Taiwan; 3 Department of Psychology, National Taiwan University, Taipei, Taiwan; 4 Division of Endocrinology and Metabolism, Department of Internal Medicine, Zhong-Xiao Branch, Taipei City Hospital, Taipei, Taiwan; 5 Department of Endocrine and Metabolism, Zhong-Xiao Branch, Taipei City Hospital, No. 87, Tongde Rd., Nangang Dist., Taipei, 11556 Taiwan

**Keywords:** Diseases, Eye diseases, Metabolic disorders

## Abstract

Effects of type 2 diabetes on achromatic and chromatic contrast sensitivity (CS) are still controversial. In this study, we aimed to investigate CS in patients without diabetic retinopathy (no-DR) and in those with non-proliferative DR (NPDR) and proliferative DR (PDR) using psychophysical methods with transient and sustained achromatic stimuli and color patches. Achromatic CS was measured with the pulsed pedestal (PP) paradigm (7, 12, and 19 cd/m^2^) and pedestal-△-pedestal (P-△-P) paradigm (11.4, 18, and 28.5 cd/m^2^). A chromatic discrimination paradigm that assesses protan, deutan, and tritan color vision was adopted. Forty-two patients (no-DR n = 24, NPDR n = 12, PDR = 6; male n = 22, mean age = 58.1 y/o) and 38 controls (male n = 18, mean age = 53.4 y/o) participated. In patients, mean thresholds were higher than in controls and linear trends were significant in most conditions. For the PP paradigm, differences were significant in the PDR and NPDR groups in the 7 and 12 cd/m^2^ condition. For the P-△-P paradigm, differences were only significant in the PDR group in the 11 cd/m^2^ condition. Chromatic contrast loss was significant in the PDR group along the protan, deutan and tritan axes. The results suggest independent involvements of achromatic and chromatic CS in diabetic patients.

## Introduction

According to the International Diabetic Federation (IDF), the number of people suffering from diabetes worldwide will rise from the current 463 million to 700 million in 2045^1^. The long-term effects of diabetes and serious consequences of poorly controlled diabetes are its accompanying complications. Among small vascular complications, the most important ones are diabetic retinopathy (DR), which is one of the leading causes of visual impairment in the age group of 20- to 70-year-olds^2^.

DR is characterized with abnormal neovascularization in the retina and iris and affects visual function. Several studies have indicated that DR is also a neurological dysfunction that occurs in combination with microvascular defects or before the onset of microvascular lesions^3,4^. Although the mechanisms of diabetic neurodegeneration and the interactions between neurodegeneration and vascular abnormalities during diabetes remain unclear, an increasing body of evidence from diabetic patients and animal models of diabetes shows that diabetes causes early retinal neurodegeneration^5,6^, and hence alters the structure and function of different types of retinal cells^3^. Whether these alterations might impair particular visual functions in diabetic patients is still not well understood^7^.

Several psychophysical studies have supported early visual functional impairment in different spatiotemporal contrast sensitivity and color vision before the onset of microvascular lesions of diabetes^7–11^. Gualtieri et al.^7^ reported achromatic contrast sensitivity (CS) reduction under the pulsed and steady pedestals paradigms in diabetic group, which was linked with losses of CS in both inferred magnocellular (M) and parvocellular (P) pathways. Impaired color vision is also thought to be an early sign of diabetic retinopathy^10^. Elevation of the arrangement errors or threshold in color arrangement tests or the Cambridge Colour Test are reported signs of early stage diabetes^10,11^. The limitation in these studies was that the influence from cataract was not well controlled. Diabetic patients are more likely to develop cataract^12^. Although cataract can decrease chromatic and achromatic CS, its effect was not well controlled in previous studies^7,10,11^.

Psychophysical assessment is usually used in clinical practice and research to assess visual functions after diabetes^13^. Although the outcomes of psychophysical evaluation are the reorganization of the retinal and brain outputs, the data based on carefully selected visual stimuli are consistent with activation of a given inferred visual pathways or brain areas^14^. Due to different responses of the dominant visual pathways to specific visual stimuli, various perceptual tasks were used in this study to selectively or preferentially at least drive the inferred pathway. Therefore, in this study, we used two psychophysical paradigms: the modified version of Pokorny–Smith’s pulsed and steady pedestals paradigm to activate the inferred M and P pathways to measure achromatic CS, and a chromatic color discrimination task to investigate chromatic contrast discrimination in type 2 diabetic patients with and without DR^15,16^.

## Materials and methods

### Participants

Approval for data collection and analysis was obtained from the Institutional Review Board of Taipei City Hospital, and all tests were conducted in accordance with the tenets of the Declaration of Helsinki. All participants gave written informed consent. Diabetic patients were prospectively recruited from the Department of Endocrine and Metabolism, Zhong-Xiao Branch, Taipei City Hospital, and had registered with the Diabetes Share Care Network. Controls were selected from outpatients without diabetes, residents living near the hospital, hospital staff, and relatives and friends of the diabetic patients.

Taiwan’s Diabetes Share Care Network (TDSCN) was established in 2001 in order to improve the quality of diabetic care. Hospitals enrolled in TDSCN programs must be certified. Diabetic patients who participate in the TDSCN receive regular biochemical checkups quarterly and ophthalmologic examinations at frequencies ranging from monthly to annually, depending on the condition of the eyes.

The inclusion criteria for the diabetic patients included: (1) type 2 diabetes; (2) age between 20 and 80 years; (3) membership in the TDSCN; and (4) receiving regular ophthalmological and biochemical examinations. The exclusion criteria were: (1) history of congenital color defects; (2) refractive errors larger than six spherical and four-cylinder diopters; (3) clinical history or evidence of ocular or neurological diseases not caused by diabetes, including trauma, multiple sclerosis, stroke, Parkinson’s disease, and Alzheimer’s disease; and (4) treatment with medications that might influence visual functioning, such as Ethambutol, Cordarone, Plaquenil, Corticosteroids, and Sabril. The inclusion criteria for the controls were: (1) fundus and visual acuity examinations in the past 6 months; and (2) age between 20 and 80 years. The exclusion criteria included: (1) history of congenital color defects; (2) refractive errors larger than six spherical and four cylinder diopters; (3) clinical history or evidence of ocular diseases, including glaucoma, laser eye surgery, age-related macular degeneration, retinitis pigmentosa, and trauma; (4) clinical history of neurological diseases that could affect CS, including multiple sclerosis, stroke, Parkinson’s disease, and Alzheimer’s disease; and (5) treatment with medications that might influence visual functioning, such as Ethambutol, Cordarone, Plaquenil, Corticosteroids, and Sabril.

### Ophthalmologic examination

In this study, each subject underwent an eye examination by an ophthalmologist using a direct ophthalmoscope to detect any signs of diabetic retinopathy, such as microaneurysms, intraretinal hemorrhages, cotton-wool spots, retinal edema, hard exudates, venous beading, neovascularization, and vitreous or preretinal hemorrhage. If any active lesions were identified, they were reported, and the severity of the diabetic retinopathy was graded based on the ophthalmoscope findings. Additionally, if macular edema was suspected, Optical Coherence Tomography (OCT) was performed to confirm the diagnosis.

### Apparatus

Stimuli were displayed on a CTX EX951F 19″ monitor driven by a MacBook Pro with an Intel HD Graphics 3000 display card. Stimuli were generated by the Psykinematix tool with the Mono 10.8 bits bit-stealing method to reach 10 bits of contrast resolution^17^. The monitor was calibrated using the X-Rite eye-one display 2 and Psykinematix software. The eye-one display 2 is a color management device. Psykinematix communicates with the eye-one display 2 to run gamma correlation and color calibration. The monitor resolution was 1280 (H) × 1024 (V), and the refresh rate was 85 Hz.

### Stimuli

First, we used the pulsed-pedestal and pedestal-△-pedestal psychophysical paradigms to assess the discrimination thresholds and achromatic contrast gain signatures of the inferred P and inferred M pathways. Then we measured the thresholds of chromatic contrast discrimination along the protan, tritan, and deutan axes^16^.

#### Pulsed-pedestal paradigm (PP paradigm)

**Figure 1:**
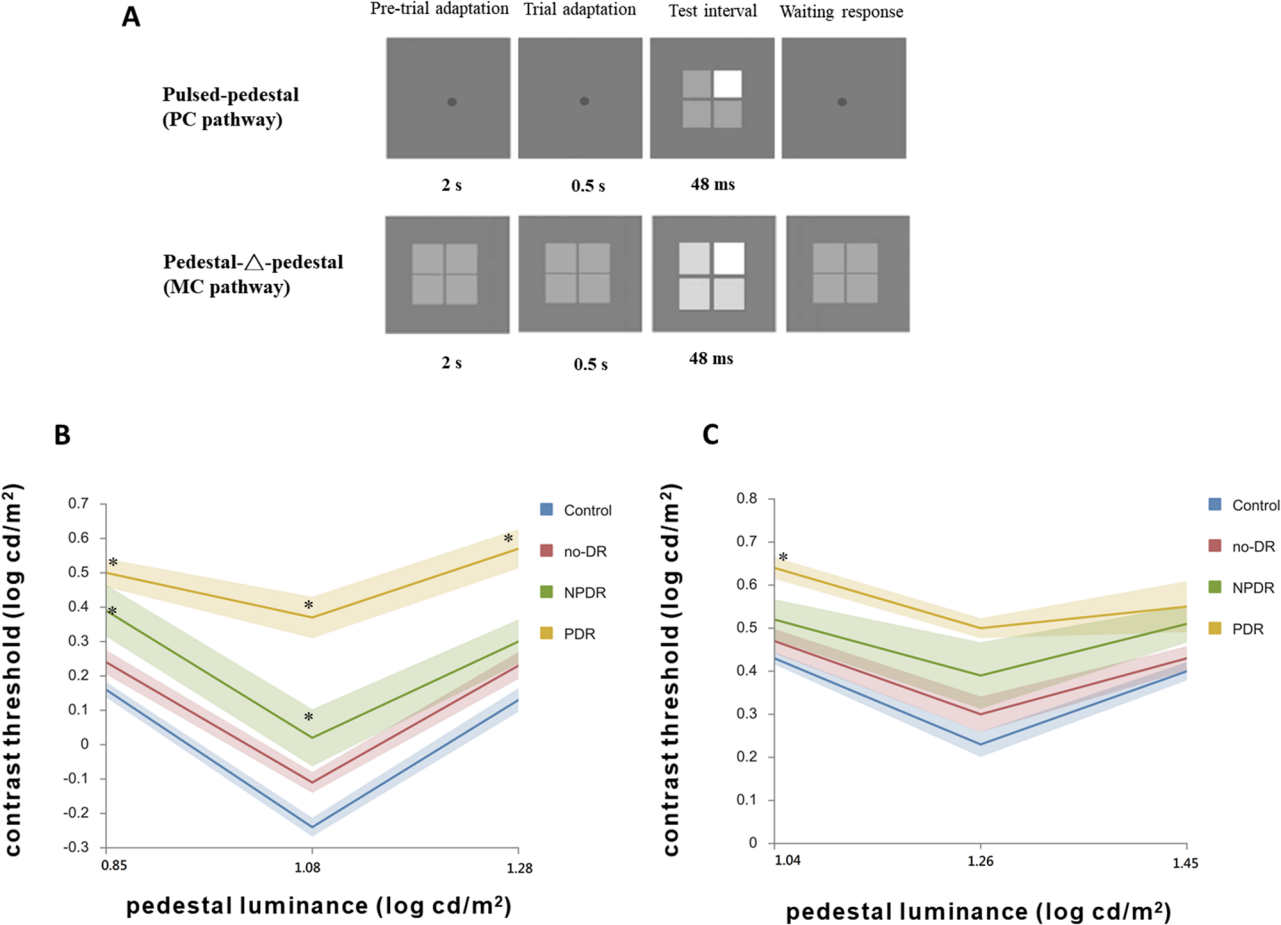
Example of the testing sequence and interactive graph of contrast thresholds (mean and standard error) from diabetic patients and controls. The pulsed-pedestal paradigm and pedestal-△-pedestal paradigm are shown in (**A**) top and (**A**) bottom, respectively. Data (**B**) is for the pulsed-pedestal paradigm and data (**C**) is for the pedestal-△-pedestal paradigm. Contrast thresholds of the no-DR, NPDR, and PDR groups were compared with the control group (*p < 0.05, ^¶^0.05 ≤ p < 0.1).

The purpose of the PP paradigm is to reveal contrast gain in the inferred P pathway^15^. Each trial of this paradigm began with adaptation to a steady and homogeneous grey background with a luminance of 12 cd/m^2^ for 2 min. Then an array of four squares of 1°*1° separated by 0.054° was presented on a constant 12 cd/m^2^ surround for 48 ms. Three grey pedestal luminance levels (7, 12, and 19 cd/m^2^) were used, and one test square was at a higher luminance than the other three (Fig. 1). Participants discriminated which square had a higher luminance than the others and remained adapted to the uniform background between trials.

#### Pedestal-△-pedestal paradigm (P-△-P paradigm)

The P-△-P paradigm was used to reveal the contrast gain in the inferred M pathway^15^. The four-square array was presented as a steady pedestal against the background during the entire session^18^. In each trial, the luminance of the four-square array was increased, with one test square (△-pedestal) having a different luminance from that of the other three for 48 ms. Participants were asked to indicate which of the four squares looked different from the other three. Three grey pedestal luminance levels (11.4, 18, and 28.5 cd/m^2^) were presented on a constant grey background with a luminance of 18 cd/m^2^ and a △-pedestal of 5.8 cd/m^2^.

#### Chromatic contrast discrimination (CCD)

**Figure 2:**
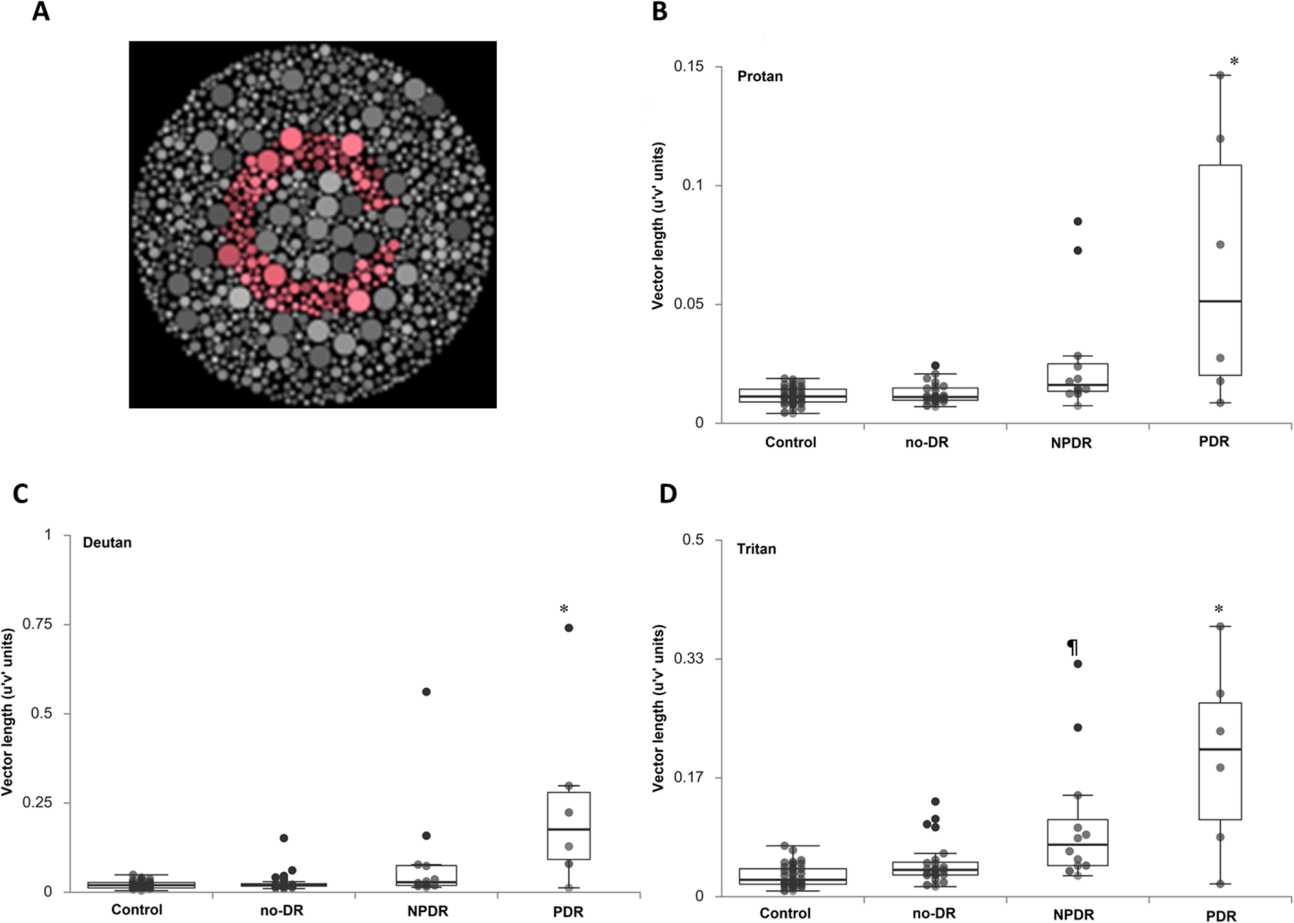
Chromatic contrast discrimination thresholds. (**A**) Example of the testing stimulus, created by the Psykinematix V1.4 (https://psykinematix.kybervision.net/). (**B**–**D**) were contrast thresholds from diabetic patients of no-DR, NPDR, and PDR groups, and controls in protan, deutan, and tritan conditions. The data are expressed in box plot (mean and standard deviation) with overlapping individual data. (*p value < 0.05, ^¶^p value < 0.1 but > 0.05 for comparisons of diabetic patients with the controls).

The CCD paradigm was meant to isolate the inferred P and K pathways. We followed the procedure of the Cambridge Colour Test developed by Regan et al.^16^. The stimulus arrays resemble the plates of a traditional pseudoisochromatic test, such as those of Stilling or Ishihara. The target is a standard Landolt-C shape differing in chromaticity from the background (Fig. 2A). The size of the Landolt-C and the background were respectively 5° and 10° of visual angle. The opening gap size of the Landolt-C optotypes was 1° of visual angle, with the gaps open in four different directions: up, right, down, and left. The chromaticity was defined in CIE 1964 *u′,v′* coordinates^19^. CCD was measured along the protan, deutan, and tritan confusion lines. The following confusion (copunctal) points were used to calculate the protan, deutan, and tritan confusion lines (neutral point: 0.1977, 0.4689; protan confusion point: 0.678, 0.501; deutan confusion point: − 1.217, 0.782; tritan confusion point: 0.257, 0.0)^20^. The stimulus was presented for 300 ms.

### Procedure

Prior to psychophysical testing, binocular visual acuity and contrast sensitivity were measured with the Vector Vision CSV-1000 chart (Vector Vision, Greenville, OH, USA) (4 rows with spatial frequencies of 3, 6, 12, and 18 cycles per degree (cpd)). The psychophysical tests were performed binocularly from a distance of 50 cm. Participants sat in a darkened room, adapted to the room’s luminance for 3 min, and then ran practice trials before the beginning of the experiment. Each participant completed 3 paradigms and 7 runs (3 for the pulsed-pedestal paradigm, 3 for the P-△-P paradigm, and 1 for the chromatic contrast discrimination paradigm). The sequences of the three paradigms were randomized. Participants were allowed to rest for 3 to 5 min between paradigms and could ask for extra time to rest if needed.

An adaptive 2 down–1 up staircase procedure for unforced-choice tasks, and the additional response alternative “I don’t know” was used for participants who had great difficulty in choosing from the four squares or directions to measure the achromatic and chromatic contrast thresholds. Participants were encouraged to identify the test square that differed from the other three and the gap direction of the target Landolt-C. Contrast was reduced after two correct responses and increased after one wrong response or a response of “I don’t know”. The decrease rate was 50% of luminance before the first reversal and 12.5% after the first reversal, and the increase rate was 25%. Each run was terminated after 6 reversals, and the mean contrast threshold was the average of the last five reversals. Auditory feedback was provided to indicate correct or incorrect responses.

### Data analysis

Descriptive statistics were used to describe participants’ characteristics and performances on the three paradigms. For comparisons of clinical characteristics among the diabetic patients and controls, one-way ANOVA was applied to analyze continuous variables, and the chi-square test was used to compare categorical variables. Because several confounding factors, either continuous (age) or categorical (sex, cataract), required adjustment to avoid bias, the performances of diabetic patient and control groups were compared by three steps. One-way ANOVA was used in the first step to determine how the different disease categories influenced the thresholds of contrast sensitivity in the different paradigms. Then, to avoid confounding bias, multiple linear regression analysis was performed after adjustments for age, cataract status, and hypertension to elucidate whether the effects of different disease categories differed in the various paradigms. Finally, linear trend was used to examine if contrast thresholds exhibited an increasing trend as diabetic retinopathy severity progressed. Pearson’s correlation was performed to measure the strengths of associations between the scores of the Early Treatment Diabetic Retinopathy Study (ETDRS) visual acuity test, and the achromatic and chromatic contrast thresholds. Statistical significance was determined at < 0.05 probability. All statistical analyses were performed in SPSS 13.0 for Windows (SPSS Inc., Chicago, IL, USA). The contrast thresholds of the three paradigms were expressed in units of log_10_ contrast sensitivity.

## Results

### Participant characteristics

Forty-four diabetic patients and 38 non-diabetic controls met the inclusion criteria and were enrolled in this study. The levels of diabetic retinopathy disease were classified into no apparent diabetic retinopathy (no-DR), NPDR, and PDR^21^. Forty-two diabetic patients (no apparent diabetic retinopathy (no-DR), n = 24; non-proliferative diabetic retinopathy (NPDR), n = 12; proliferative diabetic retinopathy (PDR), n = 6) and 38 controls finished all the tests. Two patients with NPDR withdrew from the assessment due to eye strain. Table 1 presents the characteristics of the participants. The visual acuities and contrast sensitivity of the diabetic group with no-DR were not significantly different from those of the control group (p = 0.52 for visual acuity, p = 0.09–0.74 for CS). In contrast, the CS at 4 spatial frequencies of the PDR group and the CS at 12 cpd of the NPDR group were significantly lower than those of controls. The results of contrast sensitivity measured with CSV-1000 are listed in Table S1. Significant differences between the diabetic patients and controls were found in age, status of hypertension, and cataracts. Age and cataract status were likely to affect performance, so adjustments for age and cataract status were made in the multiple regression analysis. Lens opacities were classified as nuclear sclerotic (NS), cortical opacity (CO), and posterior subcapsular cataract (PSC), and graded with the Lens Opacities Classification System II (LOCS II) in all participants. The percentage of cataract was 78.6% in the diabetic group and 13.2% in the control group. The types of cataract were NS (81.8%), NS plus PSC (9.1%), NS plus CC (3.0%), and PSC (6.1%). The impact of cataract on visual function was further classified into three groups: normal if there were no signs of cataract; mild if the opacity was equal to or less than grade three NS or grade CO (NS: 1+ to 3+, NS: 1+ to 3+ & CO: 1+ to 3+); and severe if more than grade three NS co-presented with grade four CO or PSC (NS: 4+ to 6+ , CO: 4+, NS: 1+ to 4+ & PSC, PSC). The percentages of normal and mild impacts were 21.4% and 61.9% in the diabetic group and 86.8% and 13.2% in controls. The biochemical data of the diabetic patients, including hemoglobin A1c, duration of diabetes, total cholesterol (T-CHO), triglyceride, low-density lipoprotein (LDL) cholesterol, high-density lipoprotein (HDL) cholesterol, glutamic-pyruvic transaminase (GPT), and creatinine, are also presented in Table 1. Those data were collected when the diabetic patients were first recruited into the Diabetes Share Care Network.

**Table 1:** Participant characteristics.

Variable	Type 2 diabetes (n = 42)	Non-diabetic Control (n = 38)	p value
Age (years)	58.1 (9.9)	53.4 (15.2)	0.01*
Sex			
Male	22 (52.4%)	18 (47.4%)	0.65
Female	20 (47.6%)	20 (52.6%)	
Hypertension			
Yes	26 (61.9%)	12 (31.6%)	< 0.01*
No	16 (38.1%)	26 (68.4%)	
Cataract			
Normal	7 16.7%)	32 (84.2%)	< 0.01*
Cataract	33 (78.6%)	5 (13.2%)	
s/p cataract surgery	2 (4.8%)	1 (2.6%)	
Lens opacity severity			
Normal	9 (21.4%)	33 (86.8%)	
Mild	26 (61.9%)	5 (13.2%)	< 0.01*
Severe	7 (16.9%)	0 (0.0%)	
Visual acuity (log MAR)	0.08 (0.21)	− 0.08 (0.11)	< 0.01*
VA of no-DR	0.01 (0.14)	–	0.52
VA of NPDR	0.13 (0.29)	–	< 0.01*
VA of PDR	0.27 (0.17)	–	< 0.01*
Diabetic retinopathy levels		–	–
No-DR	24 (57.1%)	–	–
NPDR	12 (28.6%)	–	–
PDR	6 (14.3%)	–	–
HbA1c (%)	7.3 (0.9)	–	–
Duration of diabetes (years)	9.7 (6.5)	–	–
T-CHO (mg/dL)	198.3 (64.3)	–	–
Triglyceride (mg/dL)	242.5 (479.5)	–	–
LDL (mg/dL)	110.3 (35.8)	–	–
HDL (mg/dL)	44.6 (12.3)	–	–
GPT (U/L)	36.3 (33.2)	–	–
Creatinine (mg/dL)	1.2 (1.3)	–	–

Continuous variables are presented as mean (SD). Categorical variables are presented as n (%), SD: standard deviation, *: indicating significant p values (p < 0.05). VA: visual acuity; no-DR: no apparent diabetic retinopathy; NPDR: nonproliferative diabetic retinopathy; PDR: proliferative diabetic retinopathy; cpd: cycles per degree; HbA1c: hemoglobin A1c; T-CHO: total cholesterol; LDT: low-density lipoprotein cholesterol, HDL: high-density lipoprotein cholesterol, GPT: glutamic-pyruvic transaminase.

### Pulsed-pedestal paradigm (PP paradigm)

Figure 1A shows examples of the stimuli of the PP and P-△-P paradigms for the diabetic patients and controls. Interactive graphs^22^ of Fig. 1B and part of Table 2 show the average contrast discrimination thresholds (unit = log cd/m^2^) and standard deviation (SD) from no-DR, NPDR, and PDR diabetic patients and controls for the PP paradigm. The minimal threshold occurred when the pedestal luminance was equal to the background luminance, so the data formed a V-shaped function of luminance. Overall, the log contrast discrimination threshold was elevated for all diabetic patients, including the no-DR group.

**Table 2:** Descriptive data and results of one-way ANOVA from diabetic patients and controls.

Paradigm	Control (n = 38)	No-DR (n = 24)	NPDR (n = 12)	PDR (n = 6)	P value
Pulsed pedestal					
7 cd/m^2^	0.157 (0.138)	0.244 (0.170)	0.387 (0.259)	0.505 (0.103)	< 0.01*
12 cd/m^2^	− 0.240 (0.165)	− 0.107 (0.149)	0.015 (0.288)	0.370 (0.147)	< 0.01*
19 cd/m^2^	0.133 (0.209)	0.233 (0.190)	0.302 (0.220)	0.570 (0.140)	< 0.01*
Pedestal-△-pedestal					
11 cd/m^2^	0.431 (0.088)	0.474 (0.138)	0.517 (0.162)	0.644 (0.062)	< 0.01*
18 cd/m^2^	0.226 (0.176)	0.296 (0.201)	0.390 (0.265)	0.498 (0.058)	< 0.01*
28 cd/m^2^	0.402 (0.132)	0.429 (0.134)	0.506 (0.148)	0.551 (0.144)	0.03*
Chromatic contrast threshold measurement					
Protan	0.012 (0.004)	0.013 (0.004)	0.027 (0.025)	0.066 (0.058)	< 0.01*
Deutan	0.021 (0.010)	0.029 (0.029)	0.088 (0.155)	0.247 (0.262)	< 0.01*
Tritan	0.029 (0.016)	0.047 (0.032)	0.103 (0.091)	0.197 (0.132)	< 0.01*

Descriptive data are presented as mean (SD). The results of the pulsed pedestal paradigm and pedestal-△-pedestal paradigm are described in units of log cd/m^2^. Data of the chromatic CS are given in CIE u′v′ units, *indicates significant p values (p < 0.05).

The mean thresholds of the PP paradigm in the diabetic groups and control group in three achromatic conditions (7, 12, and 19 cd/m^2^) are shown in Table 2. The significance levels from the multiple linear regression analysis after adjustment for age, hypertension status, and three cataract statuses (no apparent cataract, mild, and severe) for comparison of the no-DR, NPDR, and PDR groups with the control group are shown in Table 3 and Fig. 1B. For the PP paradigm, significant differences were observed in the PDR group tested with all three conditions and in the NPDR group tested with the 7 cd/m^2^ and 12 cd/m^2^ pedestal contrast conditions.

**Table 3:** Multiple linear regression analysis of the contrast discrimination thresholds in three paradigms.

Group comparison	Paradigm				Stimulus condition					
		β	SE	*P* value	β	SE	*P* value	β	SE	*P* value
Control	Pulsed pedestal	7 cd/m^2^			12 cd/m^2^			19 cd/m^2^		
No-DR		0.041	0.049	0.406	0.066	0.053	0.216	0.018	0.054	0.75
NPDR		0.167	0.063	0.010	0.162	0.069	0.020	0.056	0.070	0.43
PDR		0.267	0.777	0.001	0.511	0.084	< 0.01*	0.311	0.086	< 0.01*
P for trend		0.089	0.023	< 0.01*	0.143	0.026	< 0.01*	0.08	0.026	< 0.01*

Control	Pedestal-△-pedestal	11 cd/m^2^			18 cd/m^2^			28 cd/m^2^		
No-DR		− 0.008	0.035	0.81	− 0.029	0.052	0.57	− 0.011	0.042	0.79
NPDR		0.017	0.045	0.71	0.029	0.067	0.67	0.052	0.055	0.35
PDR		0.138	0.056	0.02*	0.120	0.082	0.15	0.099	0.067	0.14
P for trend		0.035	0.017	0.04*	0.034	0.025	0.17	0.033	0.020	0.11

Control	Chromatic CS	Protan			Deutan			Tritan		
No-DR		− 0.005	0.006	0.35	− 0.016	0.028	0.58	− 0.011	0.014	0.42
NPDR		0.007	0.007	0.34	0.037	0.036	0.31	0.035	0.018	0.05^¶^
PDR		0.044	0.009	< 0.01*	0.191	0.045	< 0.01*	0.116	0.022	< 0.01*
P for trend		0.011	0.003	< 0.01*	0.050	0.014	< 0.01*	0.033	0.007	< 0.01*

Control	Pedestal-△-pedestal	11 cd/m^2^			18 cd/m^2^			28 cd/m^2^		
No-DR		− 0.008	0.035	0.81	− 0.029	0.052	0.57	− 0.011	0.042	0.79
NPDR		0.017	0.045	0.71	0.029	0.067	0.67	0.052	0.055	0.35
PDR		0.138	0.056	0.02*	0.120	0.082	0.15	0.099	0.067	0.14
P for trend		0.035	0.017	0.04*	0.034	0.025	0.17	0.033	0.020	0.11

Control	Chromatic CS	Protan			Deutan			Tritan		
No-DR		− 0.005	0.006	0.35	− 0.016	0.028	0.58	− 0.011	0.014	0.42
NPDR		0.007	0.007	0.34	0.037	0.036	0.31	0.035	0.018	0.05^¶^
PDR		0.044	0.009	< 0.01*	0.191	0.045	< 0.01*	0.116	0.022	< 0.01*
P for trend		0.011	0.003	< 0.01*	0.050	0.014	< 0.01*	0.033	0.007	< 0.01*

Data of the controls were used as the baseline for comparison with those of the diabetic patients with no-DR, NPDR, and PDR.

*Indicates significant p values (p < 0.05), ^¶^indicates marginally significant p values (0.05 ≤ p < 0.1).

In addition, significant linear trends from the multiple linear regression analysis within the control, no-DR, NPDR, and PDR groups were observed for all testing conditions (p for trend < 0.05).

The results of correlation analysis demonstrated significantly moderate correlation between the visual acuity and contrast threshold of the PP paradigm (r = 0.68, 0.69 and 0.63 for 7, 12, and 19 cd/m^2^, respectively).

### Pedestal-△-pedestal paradigm (P-△-P paradigm)

Figure 1C and part of Table 2 show the average contrast discrimination thresholds and standard deviation (SD) for the no-DR, NPDR, and PDR diabetic patients and controls under the P-△-P paradigm. The minimal threshold occurred when the pedestal luminance was equal to the background luminance, so the data formed a V-shaped function of luminance. Overall, the log contrast discrimination threshold was elevated for all diabetic patients, including the no-DR group.

The mean thresholds of the P-△-P paradigm in the diabetic groups and control group in three achromatic conditions (11, 18, and 28 cd/m^2^) are listed in Table 2. The significance levels from the multiple linear regression analysis after adjustments for age, hypertension status, and cataract status for comparison of the no-DR, NPDR, and PDR groups with the control group are shown in Table 3 and Fig. 1C. For the P-△-P paradigm, significant difference was observed only in the PDR group tested in the 11 cd/m^2^ condition (Table 3). Significant linear trends from the multiple linear regression analysis within the control, no-DR, and NPDR groups were also only observed in the 11 cd/m^2^ condition (p for trend < 0.05).

Only weak to moderate correlations were found between the visual acuity and contrast threshold in the P-△-P conditions for all pedestal luminances (r = 0.49 for 11 cd/m^2^, 0.60 for 18 cd/m^2^, and r = 0.30 for 28 cd/m^2^).

### Chromatic contrast discrimination (CCD)

Figure 2A shows an example of the stimulus of the CCD paradigm. Figure 2B, a box plot with overlapping individual data^23^, shows the results of chromatic contrast discrimination in the CIE u′v′ color space. The original contrast discrimination threshold was converted into u′v′ units by multiplying the contrast by the vector length between the neutral and copunctal points. The results of one-way ANOVA indicated significant (along the protan and deutan axes) and marginally significant (tritan axis) differences in the mean contrast thresholds between the control, no-DR, NPDR, and PDR groups (Table 2). The significance levels for the multiple linear regression analysis after adjustment for age and cataract status are shown in Fig. 2, with * indicating a p value < 0.05 and ¶ indicating marginal significance (0.05 ≤ p < 0.1) for comparisons of the no-DR, NPDR, and PDR groups with the control group. Significant chromatic contrast loss was observed in the PDR group along the protan, deutan and tritan axes. In addition, a marginal significance level was also found in the NPDR group along the tritan axis (Table 3). There were significant between-group differences (Table 2), and significant linear trends from the multiple linear regression analysis were observed across all conditions (p for trend < 0.05) (Table 3).

## Discussion

In this study, we conducted three psychophysical experiments to evaluate achromatic and chromatic CS in diabetes without and with DR. The P-△-P and PP paradigms were used to measure achromatic CS, in which the patterns of contrast saturation differed. Although our results did not reveal significant differences between the no-DR group and controls, the data demonstrated that diabetic patients in the NPDR group exhibited more significant losses in CS in the inferred P pathway than in the inferred M pathway. Chromatic CS was significantly reduced in the PDR diabetic group relative to the control group for stimuli along the protan, deutan, and tritan color axes. However, significant linear trends in threshold elevations were found in achromatic and chromatic contrast discrimination.

Some of our results are consistent with a previous finding by Gualtieri et al. that patients with diabetic retinopathy tend to have higher contrast sensitivity loss^7^. In our study, significant linear trends in the three tasks were observed in the no-DR, NPDR, and PDR diabetic groups. Functional defects in achromatic contrast modulation were related to severity of retinal damage in diabetic patients. Most data of this study support that diabetic patients with PDR consistently showed significant functional losses in the inferred M and P pathways. Although no significant difference was found in the NPDR group in the 19 cd/m^2^ pulsed pedestal condition, this may have resulted from the small sample size of the NPDR group. However, in our study, no significant contrast sensitivity loss was observed in the inferred pathway in the PDR group in the 18 and 28 cd/m^2^ conditions, although several studies have indicated that diabetic patients without apparent retinopathy show perceptual deficits after variation of temporal parameters^7,24^. Our results demonstrate that diabetic patients tend to have impaired function in the inferred P pathway. This finding was consistent with the study by Gualtieri et al.^7^. However, in contrast to the findings of Gualtieri et al., there was no significant difference between no-DR diabetic participants and controls. The reason may be the small difference between the no-DR and control groups in this study, such as the lack of a significant difference in visual acuity and contrast sensitivity function (Table S1). The paradigms used in this study may not be sufficiently sensitive for detection of the CS difference or for application to early neurodegeneration diagnosis in such diabetic patients.

The results indicating impaired inferred P pathway processing were consistent with the correlation analysis between the values of contrast threshold and visual acuity across all pulsed pedestal conditions. Lesions specific to the inferred P pathway have been shown to decrease the visual acuity of macaque monkeys^25^. We found relatively higher correlations between diabetic patients’ visual acuities and their thresholds in the PP paradigm, but not in the P-△-P paradigm. These significant relationships might indicate a differential loss in the inferred P and M pathways or a differential loss of ganglion cell function^26–28^.

Several studies have indicated a dramatically increased prevalence of color vision impairment in diabetic patients without diabetic retinopathy^11,29^. Some studies have found diffuse loss in color perception^11,30^, and some have shown early dominant loss in blue–yellow (tritan) discrimination^31^. In our study, diffuse losses in protan, deutan, and tritan color vision were observed in no-DR diabetic patients. However, the predominant loss was found to occur along the tritan measure. These findings are consistent with previous studies demonstrating selective loss of S cones due to diabetes^32–34^. However, it may be suggested that this effect may also result from lens yellowing. There was a dramatically higher prevalence of cataract in diabetic patients (in this study, diabetic group: 78%, controls: 13%). Even though the variable of cataract status was considered in the multiple linear regression analysis, the overall results of the no-DR, NPDR, and PDR groups also showed predominant tritan color vision loss. However, it is not easy to completely exclude the influence of lens yellowing. In addition, the elevation of the protan and deutan color contrast thresholds was consistent with significant deficits in achromatic contrast sensitivity in the PP paradigm.

Feitosa-Santana, et al. indicated that elevation of the tritan threshold in the Cambridge Colour Test was found to correlate with the blood sugar level^10^. The diabetic patients in our study were all recruited in the TDSCN program and received standard care to optimize their blood sugar, blood pressure, and lipid control. If their blood sugar control was poor (A1c > 7%), different types of oral anti-diabetic drugs, including metformin, DPP-4i, SGLT-2i, TZDs, Sus, GLP-1RA, and insulin, were used to lower their blood glucose. Our study found that over 60% of the diabetic patients achieved their goal of A1c < 7%, indicating that they were well-controlled diabetic patients. This may explain the lack of significant differences between normal controls and diabetics with no-DR.

In our experience, it is hard to require all participants, and especially diabetic participants, to entirely follow the forced choice rule during measurement. Therefore, unforced-choice tasks with the additional response alternative “I don’t know” were adopted in this study. Although we informed the participants that they could select “I don’t know”, we also encouraged participants to choose one of the four squares or directions and not to select the “I don’t know” response as much as possible. Actually, few participants selected it. We rechecked the responses on which diabetic patients and controls selected the “I don’t know” response among all the testing trials. In fact, few participants selected this response in the P-△-P paradigm in the 28.5 cd/m^2^ condition or in the CCD paradigm. None of the controls chose this response.

The main limitation of this study is that only limited psychophysical conditions were adopted to assess the achromatic and chromatic CS. This small set of conditions may influence the degree of precision and accuracy of characterizing and quantifying early visual deficits in diabetic patients. However, the purpose of this study was to modify and translate current reliable paradigms to clinical use to aid in the detection and diagnosis of early visual impairment, for which a time-consuming assessment would be unsuitable. In this context, modified psychophysical measurement that directly examines the relationships between visual stimuli and either sensation or perception will have potential as a clinical assessment tool. Another limitation is the sample size of diabetic patients with NPDR. Although the small sample size affected the significance level, NPDR patients were found to have apparent CS deficits in the pulsed pedestal paradigm of the 7 cd/m^2^ condition.

## Conclusion

The results of this study suggest different and independent involvements of different psychophysical conditions in diabetic patients. The neural pathways involved with the PP and CCD paradigms seem to be more susceptible to diabetes. Our findings are also consistent with other higher-level visual functions, such as visual acuity, and with the responses from retinal physiology or electrophysiological measures. Therefore, reliable psychophysical assessment appears to be an appropriate tool for use in the early identification of visual deficits in diabetic patients. Further studies to investigate the reliability of these tests and relations between contrast thresholds with biochemical data, stages of retinopathy, and retinal structure images, such as optical coherence tomography, are suggested.

## Supplementary Information

Supplementary Table S1.

## Data Availability

The datasets used and/or analysed during the current study available from the corresponding author on reasonable request.
