# Supplementary material for: Achromatic and chromatic contrast discrimination in patients with type 2 diabetes
Source: Sci Rep. 2023 May 8;13:7420. doi: 10.1038/s41598-023-34407-1 (PMC10167204; doi:10.1038/s41598-023-34407-1)
Supplement: Supplementary file 1 — Supplementary Table S1. [file 41598_2023_34407_MOESM1_ESM.pdf]

Achromatic and Chromatic Contrast Discrimination in Patients with Type 2 Diabetes

Li-Ting Tsai[1,2], Chien-Chung Chen[3], Chiun-Ho Hou[2], Kuo-Meng Liao[4*]

Article Type: Research Article

Short Title: Contrast Discrimination and Type 2 Diabetes

[1] School of Occupational Therapy, College of Medicine, National Taiwan University, Taipei, Taiwan.

[2] Department of Ophthalmology, National Taiwan University Hospital, Taipei, Taiwan College of Medicine, National Taiwan University, Taipei, Taiwan.

[3] Department of Psychology, National Taiwan University, Taipei, Taiwan.

[4*] Division of Endocrinology and Metabolism, Department of Internal Medicine, Zhong-Xiao branch, Taipei City Hospital, Taipei, Taiwan. Email: kuomeng@gmail.com

Correspondence to:

Kuo-Meng Liao, Department of Endocrine and Metabolism, Zhong-Xiao branch, Taipei City Hospital, No. 87, Tongde Rd., Nangang Dist., Taipei, 11556, Taiwan.

Tel: 886-2-27861288, ext: 1742; Fax: 886-2-87858371

Table S1 Multiple linear regression analysis of the contrast sensitivity thresholds in four spatial frequency conditions after adjusting for age and cataract effects

Group	Stimulus Condition											
Comparison	β	SE	*P* value	β	SE	*P* value	β	SE	*P* value	β	SE	*P* value
control		sf 3			sf 6			sf 12			sf 18	
no-DR	-0.137	0.079	0.087	-0.033	0.097	0.735	-0.077	0.103	0.454	-0.057	0.091	0.533
NPDR	-0.107	0.100	0.289	-0.217	0.126	0.089	-0.319	0.134	0.019*	-0.141	0.119	0.240
PDR	-0.335	0.122	0.008*	-0.567	0.153	< 0.001*	-0.867	0.163	< 0.001*	-0.862	0.145	<0.001*

Data of the controls (n=38) were used as the baseline for comparison with those of the diabetic patients with no-DR (n=21), NPDR (n=10), and PDR (n=4). *: indicates significant p values ($p < 0.05$). sf: spatial frequency. The data of contrast sensitivity thresholds in log scale were obtained from the CSV-1000 test.
